# Supplementary material for: An Improved Chromosome-Level Genome Assembly of the Firefly Pyrocoelia pectoralis
Source: Insects. 2024 Jan 8;15(1):43. doi: 10.3390/insects15010043 (PMC10816139; doi:10.3390/insects15010043)
Supplement: Supplementary file 1 [file insects-15-00043-s001.zip › insects-2740815-supplementary.pdf]

Supplementary Material

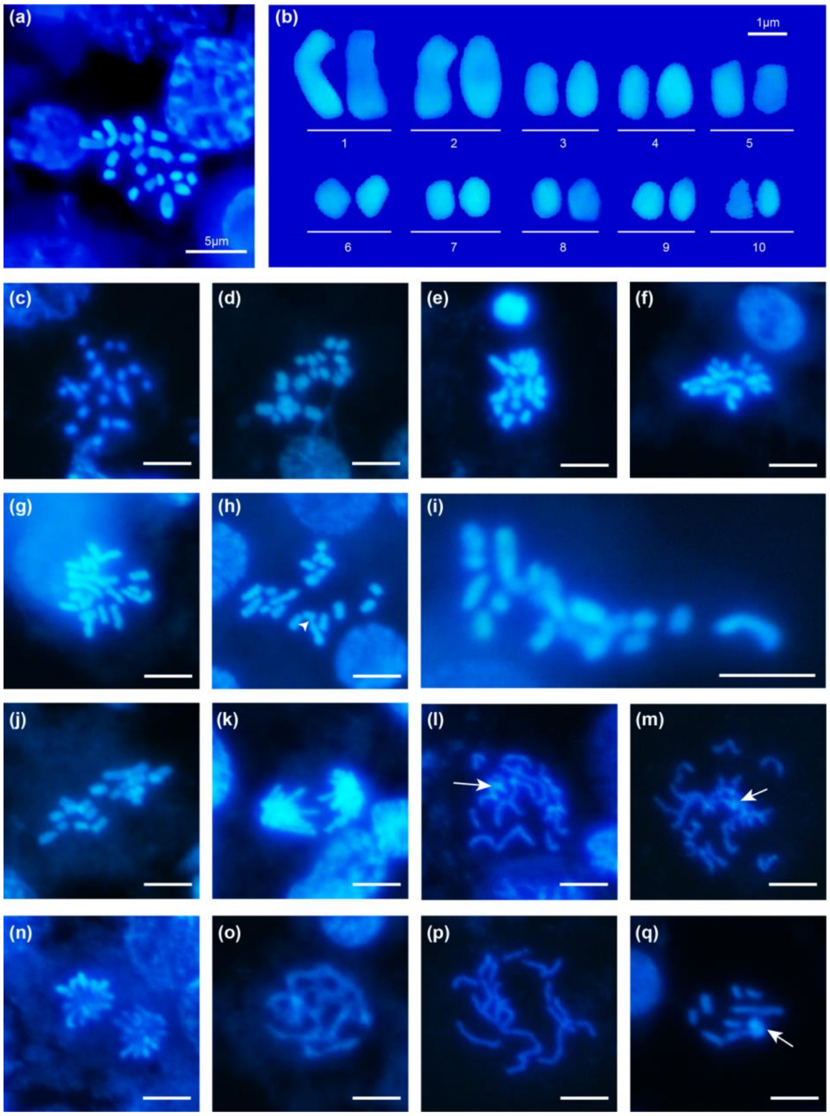

Figure S1. Karyotype analysis of *Pyrocoelia pectoralis*. (a) Spermatogonial metaphase stained with DAPI, 2n=20, Scale bar, 5μm. (b) Karyogram, Scale bar, 1μm. (c-g) Mitotic metaphase of *P. pectoralis*. (h-k) the chromosome are pulled apart and move to the opposite ends of the cell, triangle indicated possible Y chromosome, which is much smaller than X chromosome, (l-n) Meiosis I of *P. pectoralis.* Arrows indicated diffuse diplotene with the condensed sex chromosome and decondensed bivalents, (o-q) Meiosis II of *P. pectoralis.* Arrow indicated the sex chromosomes at the top very condensed. Scale bar, 5μm.

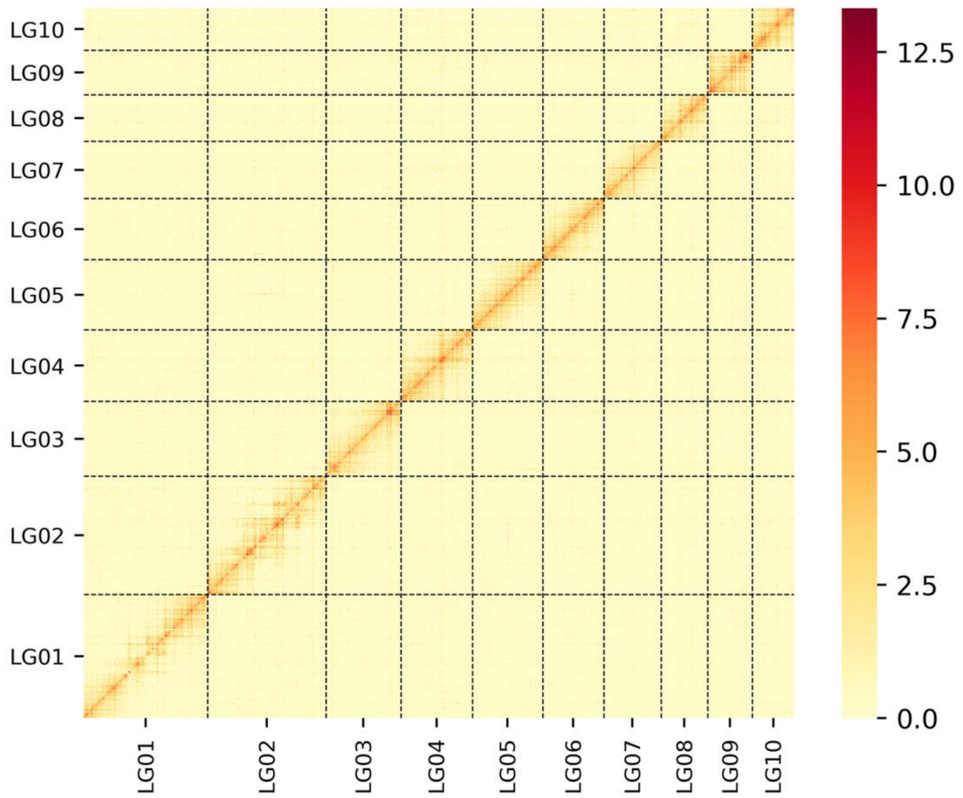

Figure S2. Hi-C contact data mapped on the updated *P. pectoralis* Hi-C genome Ppec-2.0, showing genome-wide all-by-all interactions

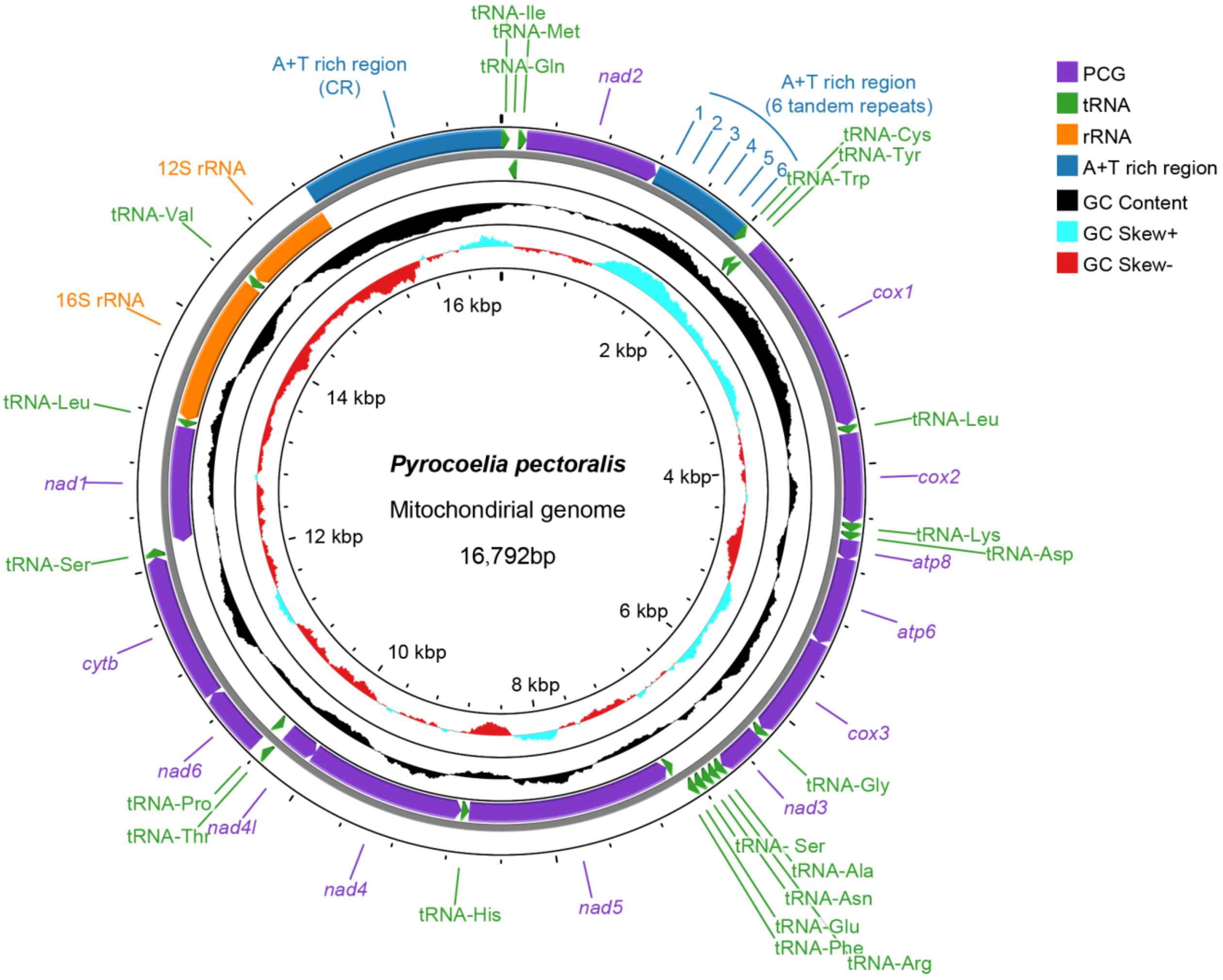

Figure S3. Graphical map of the complete mitochondrial genome of *P. pectoralis*. Drawn to scale as indicated by the innermost circle. The inner circle indicates the GC skew (using a red and blue sliding window, indicating its positive and negative values respectively). The middle circle indicates the GC content. The outer circle indicates gene arrangement, genes encoded by the direct strand were shown outside, and encoded by the reverse strand inside the outermost circle respectively (arrows indicate the direction of gene transcription). The statistic was based on a 500bp window.

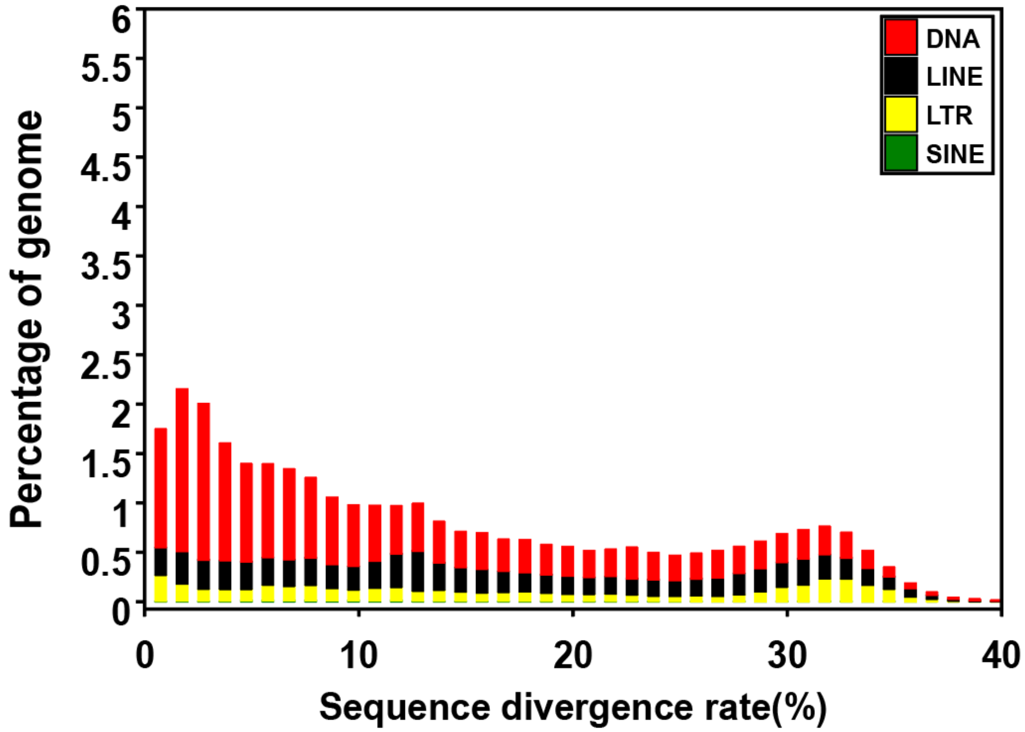

Figure S4. Distribution of divergence rate for the four types of TE in *P. pectoralis* Hi-C genome Ppec-2.0

(a)

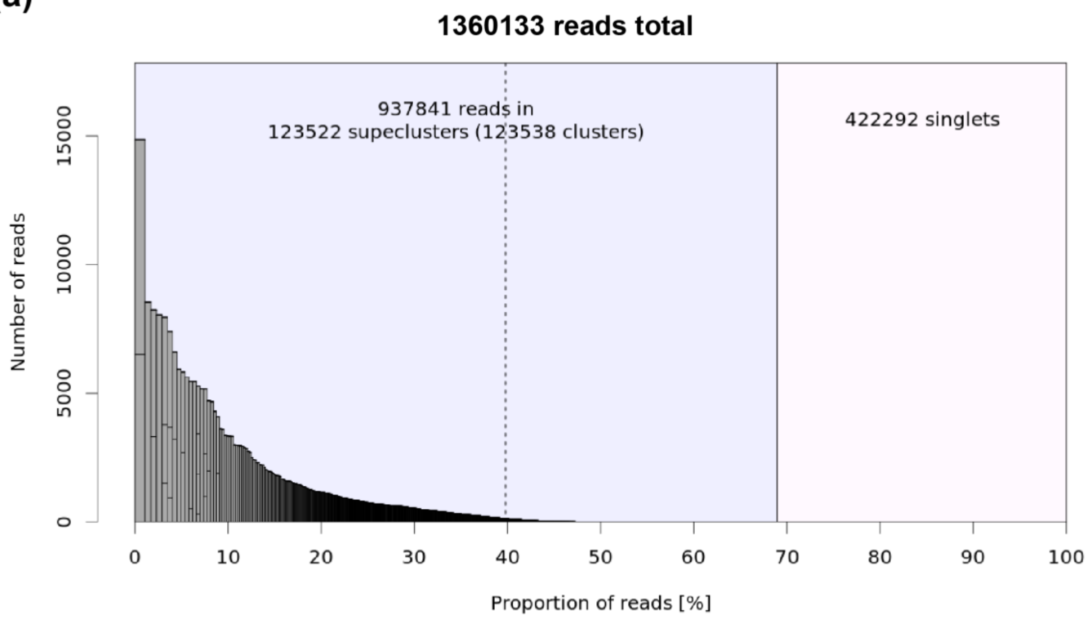
1360133 reads total
937841 reads in
123522 supeclusters (123538 clusters)
422292 singlets
Number of reads
0
5000
10000
15000
0
10
20
30
40
50
60
70
80
90
100
Proportion of reads [%]

(b)

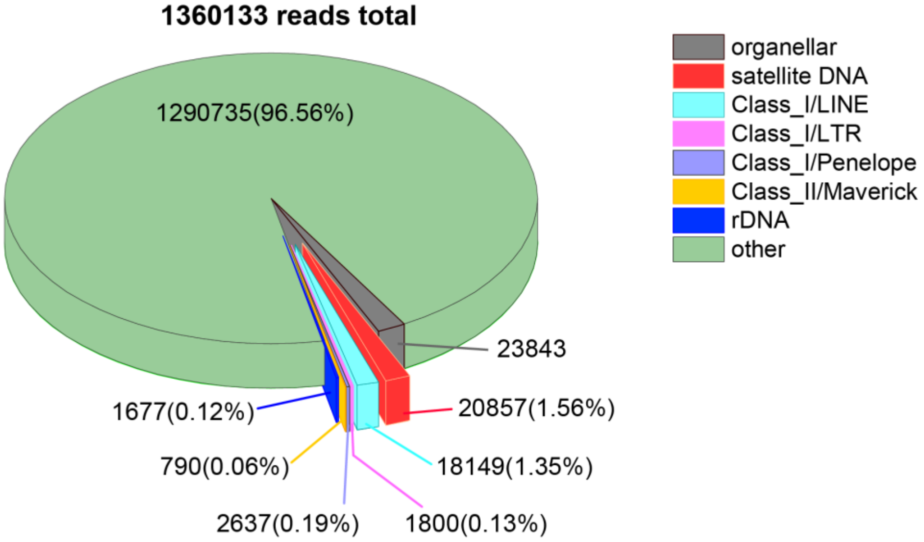
1360133 reads total
organellar
satellite DNA
Class_I/LINE
Class_I/LTR
Class_I/Penelope
Class_II/Maverick
rDNA
other
1290735(96.56%)
23843
20857(1.56%)
18149(1.35%)
1800(0.13%)
2637(0.19%)
790(0.06%)
1677(0.12%)

Figure S5. De novo identification of satellite DNAs in the sequenced genomes of *P. pectoralis* using the RepeatExplorer. (a) Graphical summary of the clustering results of *P. pectoralis* Illumina unassembled sequences. Bars represent superclusters, with their heights and widths corresponding to the numbers of reads in the superclusters (y-axis) and to their proportions in all analyzed reads (x-axis), respectively. Rectangles inside the supercluster bars represent individual clusters. If the filtering of abundant satellites was performed, the affected clusters are shown in green, and their sizes correspond to the adjusted values. Blue and pink background panels show proportions of reads that were clustered and remained single, respectively. Top clusters are on the left of the dotted line. (b) Repeat annotation summary the automatic annotations of clusters.

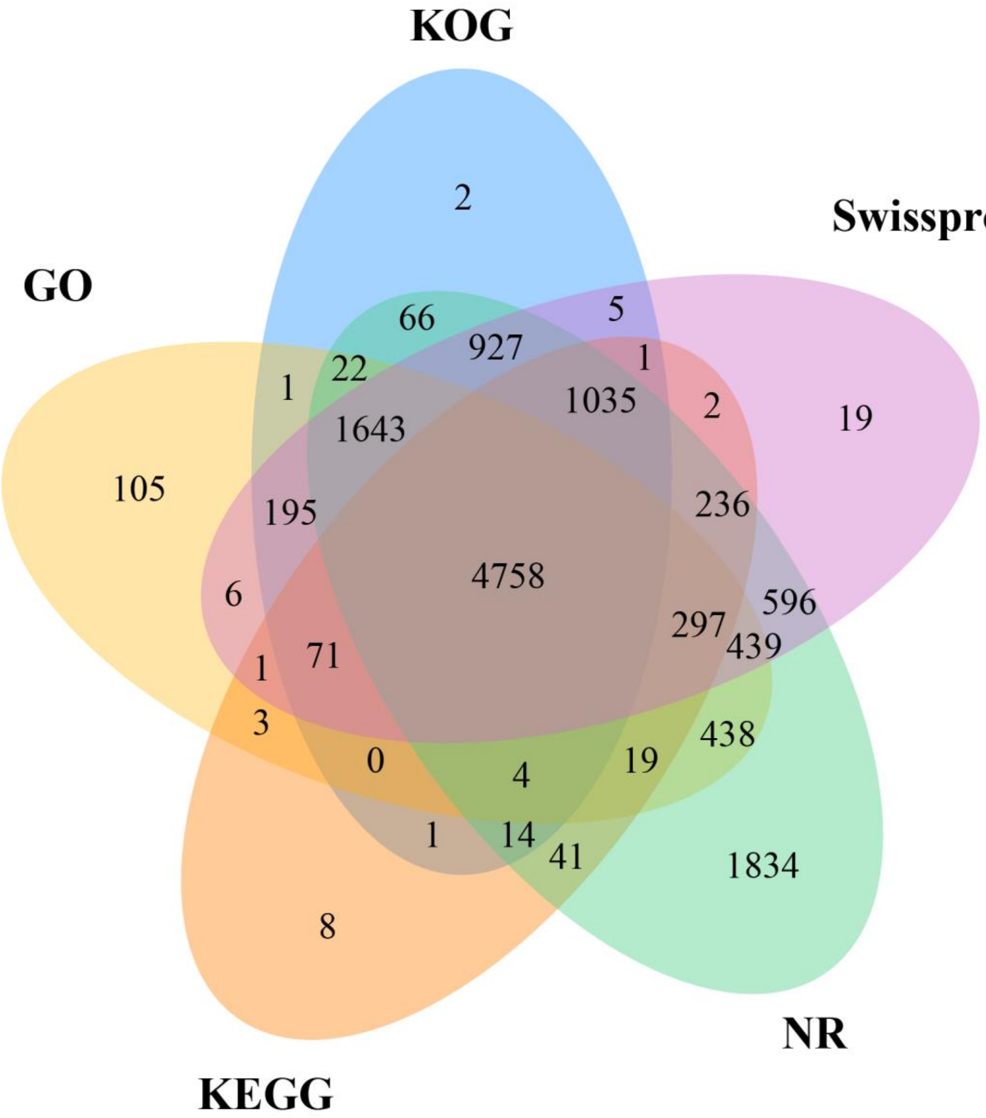

Figure S6. Venn diagram of gene function annotation by Swissprot, KEGG, GO, NR and KOG

(a)

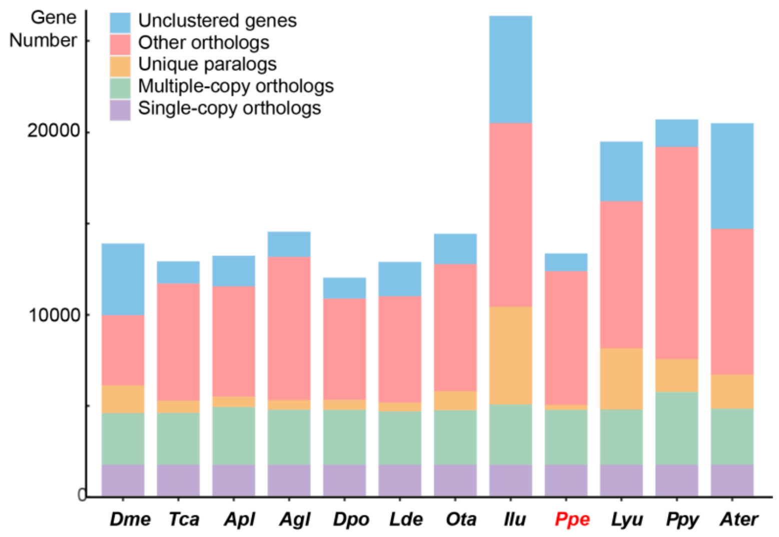

(b)

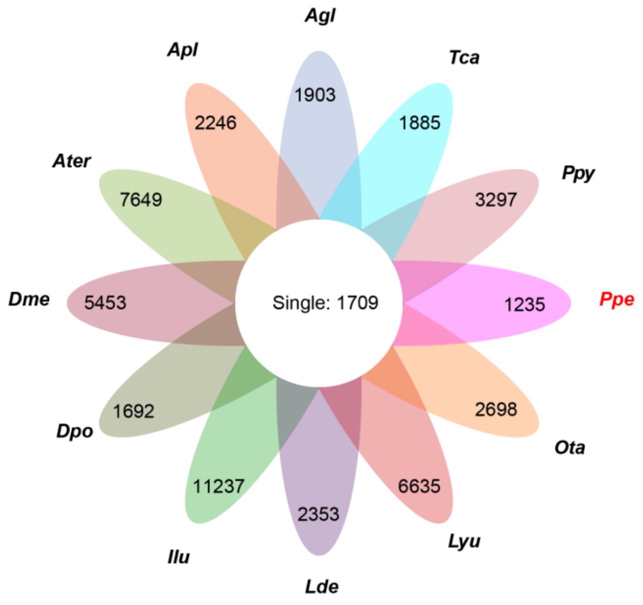

Figure S7. Orthogroup (OGs) clustering analysis of peptides in 12 insects. (a) the multiple-copy orthologs are orthologs that have multiple copies in one species, the single-copy orthologs are orthologs that have only a single copy in one species, the other orthologs are the remainders of the orthologs; the unclustered genes are genes that have no homology with others, and the unique paralogs are genes that only exist in one specific species. Dme, *Drosophila melanogaster;* Tca, *Tribolium castaneum;* Apl, *Agrilus planipennis;* Agl, *Anoplophora glabripennis;* Dpo, *Dendroctonus ponderosae;* Lde, *Leptinotarsa decemlineata;* Ota, *Onthophagus taurus;* Ilu, *Ignelater luminosus;* Ppe, *Pyrocoelia pectoralis;* Lyu, *Lamprigera yunnana*; Ppy, *Photinus pyralis;* Ate, *Abscondita terminalis*; (b) Flower plot of gene clusters for 12 genomes. Single refers to universal single-copy genes; the petals showing numbers of species-specific orthologous genes in each species.

Table S1. Table summary of assembly using PacBio data [12] and Hi-C data for *P. pectoralis*

Stat Type	Original de novo genome [12]*		original genome Ppec-1.0 [12]**		Hi-C genome Ppec-2.0***	
	Contig. Length (bp)	Contig. Number	Scaffold Length (bp)	Scaffold Number	Scaffold Length (bp)	Scaffold Number
N50	2,316,748	136	3,035,809	79	52,870,853	4
N60	1,749,500	193	2,521,720	106	52,416,343	5
N70	1,199,028	271	1,842,775	143	45,666,288	6
N80	714,909	394	1,377,538	190	33,942,204	8
N90	161,781	689	813,338	261	31,604,859	9
Longest	13,688,299	1	13,688,299	1	90,690,252	1
Total	1,119,821,639	3,517	760,416,098	474	532,250,877	363
Length>=1kb	1,119,806,285	3,493	760,416,098	474	532,242,980	350
Length>=2kb	1,119,729,364	3,444	760,323,807	464	532,229,674	341
Length>=5kb	1,119,165,291	3,283	759,623,670	444	532,102,132	305

*The Original de novo genome is the primary assembly genome by de novo assembly of PacBio long reads;

**The original genome Ppec-1.0 is the filtered assembly genome by PacBio and Illumina HiSeq X Ten platforms selectively remove alternative heterozygous contigs;

***The Hi-C genome Ppec-2.0 is the improve genome assembly by Hi-C technology.

Table S2. Table summary of original genome Ppec-1.0 contig sequences alignment information of *P. pectoralis*

Type	Contig Number	Contig Number ratio(%)	Contig Length(bp)	Contig Length ratio(%)
Bacteria	2,413	85.54	113,250,053	17.53
Metazoa	147	5.21	526,185,213	81.45
Mitochondrion /Chloroplast	40	1.42	411,688	0.06
Viruses	4	0.14	76,143	0.01
Fungi	1	0.04	12,945	0.00
Nohit	216	7.66	6,065,664	0.94
Total	2,821	100.00	646,001,706	100.00

Table S3. Evaluation on the completeness of *P. pectoralis* genome assembly and proteins for the predicted genes by BUSCO 5.1.3

Type	original genome Ppec-1.0 [12]		original genome Ppec-1.0 predicted proteins[12]		Hi-C genome Ppec-2.0		Hi-C genome Ppec-2.0 predicted proteins	
	Number	Percent	Number	Percent	Number	Percent	Number	Percent
Complete BUSCOs (C)	1355	99.1%	1302	95.2%	1295	94.7%	1241	90.8%
Complete and single-copy BUSCOs (S)	831	60.8%	819	59.9%	1259	92.1%	1203	88.0%
Complete and duplicated BUSCOs (D)	524	38.3%	483	35.3%	36	2.6%	38	2.8%
Fragmented BUSCOs (F)	5	0.4%	13	1.0%	8	0.6%	12	0.9%
Missing BUSCOs (M)	7	0.5%	52	3.8%	64	4.7%	114	8.3%
Total BUSCO groups searched (insecta_odb10)	1367	100%	1367	100%	1367	100%	1367	100%

Table S4. Table summary of quality assessment of Hi-C data

Stat Type	Hi-C unassembled short read
Raw Paired-end Reads	594,790,586
Clean Paired-end Reads	577,074,474
Clean Bases(bp)	83,423,173,027
Clean Paired-end Reads Rate (%)	97.02
Clean Q30 Bases Rate (%)	93.23
Valid Interaction Pairs	33,404,550
Unique Map Reads	74.76%

Table S5. Hi-C Chromosome assembly result

Chr	Length(bp)	Scaffold Number
LG01	90,683,752	66
LG02	86,755,173	52
LG03	55,208,106	32
LG04	52,868,053	29
LG05	52,414,443	20
LG06	45,664,588	18
LG07	41,957,738	24
LG08	33,939,104	32
LG09	31,600,759	42
LG10	30,862,095	22
Total	521,953,811	337

Table S6. Distribution of 98,800 repeat sequences in the *P. pectoralis* Hi-C genome Ppec-2.0 based on repeat type

Class	Order	Super family	Number of elements	Length of sequence (bp)	Percentage of sequence (%)
Class I			261,291	67,737,531	12.73
	LINE		163,476	44,613,355	8.38
		Unknown	60,601	14,590,546	2.74
		R1	3,990	1,180,868	0.22
		L2	15,692	4,276,543	0.8
		RTE-BovB	4,492	1,270,300	0.24
		RTE-X	12,541	5,842,032	1.1
		Penelope	15,445	3,957,169	0.74
		Dong-R4	5,144	2,901,787	0.55
		R1-LOA	26,352	6,120,184	1.15
		CR1	6,726	1,513,233	0.28
		I	2,799	611,641	0.11
		I-Jockey	5,874	1,871,210	0.35
		Other	3,820	477,842	0.09
	LTR		82,437	22,054,471	4.14
		Unknown	53,458	9,648,871	1.81
		Gypsy	12,564	6,312,796	1.19
		Copia	6,982	2,700,543	0.51
		Pao	7,011	3,234,096	0.61
		Other	2,422	158,165	0.03
	SINE		15,378	1,069,705	0.2
		Unknown	15,220	1,062,245	0.2
		Other	158	7,460	0
Class II			578,268	138,799,300	26.08
	DNA		412,713	96,920,898	18.21
		Unknown	275,690	62,284,215	11.7
		TcMar-Mariner	28,793	5,882,870	1.11
		TcMar-Tc1	46,561	12,673,692	2.38
		hAT-Tip100	8,947	1,667,325	0.31
		hAT-Charlie	3,791	560,519	0.11
		Maverick	3,873	1,945,536	0.37
		Academ-1	16,982	5,514,932	1.04
		TcMar-m44	1,670	585,731	0.11
		PiggyBac	2,189	782,647	0.15

		P	2,411	820,624	0.15
		Other	21,806	4,202,807	0.79
	MITE		160,831	41,022,547	7.71
		Unknown	160,831	41,022,547	7.71
	RC		4,724	855,855	0.16
		Helitron	4,724	855,855	0.16
Total TEs			839,559	206,536,831	38.8
Tandem Repeats			37,180	4,894,814	0.92
	tandem repeat		20,713	4,661,540	0.88
	SSR		16,467	233,274	0.04
Unknown			107,841	15,829,691	2.97
Other			1,678	199,573	0.04
Simple repeats			1,701	231,388	0.04
Low complexity			41	5,366	0
**Total Repeats**			**988,000**	**227,697,663**	**42.78**

Table S7. Transposable elements (TEs) content in the assembled genomes of fireflies *Lamprigera yunnana, Abscondita terminalis, Aquatica lateralis, Photinus pyralis* and *P. pectoralis*

Species	Type	Length (bp)	% in genome
L. yunnana	DNA	367,551,516	34.91
	LINE	162,115,182	15.40
	SINE	2,204,348	0.21
	LTR	1,766,904	0.17
	Other	1,382	0.00
	Unknown	0	0
	**Total repeat**	**688,292,463**	**65.37**
A. terminalis	DNA	88,325,208	17.62
	LINE	49,456,973	9.86
	SINE	715,335	0.14
	LTR	4,638,621	0.93
	Other	60	0.00
	Unknown	0	0
	**Total repeat**	**169,247,169**	**33.76**
A. lateralis	DNA	134,817,777	14.84
	LINE	67,707,232	7.45
	SINE	1,184,109	0.13
	LTR	2,704,957	0.29
	Other	177	0.00
	Unknown	0	0
	**Total repeat**	**249,489,409**	**27.46**
P. pyralis	DNA	118,270,813	25.08
	LINE	40,283,641	8.54
	SINE	1,748,154	0.37
	LTR	9,967,599	2.11
	Other	0	0
	Unknown	9,853,553	2.09
	**Total repeat**	**218,446,960**	**46.33**
P. pectoralis	DNA	96,920,898	18.21
	LINE	44,613,355	8.38
	SINE	1,069,705	0.2
	LTR	22,054,471	4.14
	Other	199,573	0.04
	Unknown	15,829,691	2.97
	**Total repeat**	**227,697,663**	**42.78**

Table S8. Gene comparison between *P. pectoralis* Hi-C genome Ppec-2.0 with other species

Gene set	Total number of genes	Average gene length (bp)	Average CDS length(bp)	Average exons number per gene	Average exon length (bp)	Average intron length (bp)
P. pectoralis	13,292	14,806	1481	5.18	285	3188
L. yunnana	19,443	16,619	1377	4.74	290	3647
A.terminalis	20,436	7,767	1483	4.77	311	1532
P. pyralis	20,646	8,113	1489	5.15	289	1595
A. planipennis	13,358	11,946	1541	5.75	268	2190
A. glabripennis	14,815	16,649	1511	5.58	271	3305
D. ponderosae	13,021	8,398	1503	6.78	221	1193
L. decemlineata	14,000	14,162	1377	5.05	272	3156
O. taurus	14,537	7,834	1523	4.54	335	1783
T. castaneum	12,863	6,999	1550	5.28	293	1273
D.melanogaster	13,918	4,597	1560	3.9	400	1047

Table S9. Summary of gene function annotation of the assembled Hi-C genome Ppec-2.0 of *P. pectoralis*

Type		Number	Percent (%)
Annotation	Swissprot	10,231	76.97
	KEGG	6,491	48.83
	KOG	8,745	65.79
	GO	8,002	60.2
	NR	12369	93.06
Total	Annotated	12,789	96.22
	Gene	13,292	-

Table S10. Annotation of non-coding RNAs in the *P. pectoralis* Hi-C genome Ppec-2.0.

Type		Copy Number	Average Length(bp)	Total Length(bp)	Percentage of sequence (%)
rRNA	18S	171	1920.46	328399	0.0617
	28S	111	5425.7	602253	0.1132
	5.8S	42	160.9	6758	0.0013
	5S	110	113.46	12481	0.0023
microRNA	snRNA	6	143.17	859	0.0002
	miRNA	54	84.83	4581	0.0009
	spliceosomal	55	143.22	7877	0.0015
	other	418	285.17	119201	0.0224
Regulatory	cis-regulatory elements	190	97.75	18573	0.0035
tRNA	tRNA	2898	75.24	218057	0.041

Table S11. Summary of gene families in 12 species

Order	Family	Species	Total genes	Single	Multi	Unique	Other	Unclustered	Family number	Unique Families number
Diptera	Drosophilidae	*D. melanogaster*	13842	1709	2,829	1,523	3851	3,930	7,513	433
Coleoptera	Tenebrionidae	*T. castaneum*	12863	1709	2,848	670	6421	1,215	9,532	200
Coleoptera	Buprestidae	*A. planipennis*	13166	1709	3,167	573	6044	1,673	8,961	187
Coleoptera	Cerambycidae	*A. glabripennis*	14487	1709	3,018	527	7857	1,376	9,887	172
Coleoptera	Curculionidae	*D. ponderosae*	11968	1709	3,015	556	5552	1,136	8,792	215
Coleoptera	Chrysomelidae	*L. decemlineata*	12829	1709	2,933	483	5834	1,870	9,049	174
Coleoptera	Scarabaeidae	*O. taurus*	14374	1709	2,997	1,039	6970	1,659	9,510	314
Coleoptera	Elateridae	*I. luminosus*	26325	1709	3,310	5,364	10069	5,873	11,232	1363
**Coleoptera**	**Lampyridae; Lampyrinae**	***P. pectoralis***	**13292**	**1709**	**3,016**	**268**	**7332**	**967**	**9,543**	**91**
Coleoptera	Lampyridae; Lampyrinae	*L. yunnana*	19433	1709	3,031	3,369	8058	3,266	9,729	431
Coleoptera	Lampyridae; Lampyrinae	*P. pyralis*	20646	1709	3,996	1,799	11644	1,498	11,196	507
Coleoptera	Lampyridae; Luciolinae	*A. terminalis*	20436	1709	3,077	1,866	8001	5,783	10,287	627

Table S12. GO and KEGG enrichment of the gene family expansion orthologous genes in *P. pectoralis* Hi-C genome Ppec-2.0

Class	ID	Description	p value	Count
KEGG	map00980	Metabolism of xenobiotics by cytochrome P450	6.79E-11	22
KEGG	map00981	Insect hormone biosynthesis	1.05E-10	15
KEGG	map00982	Drug metabolism - cytochrome P450	3.71E-08	18
KEGG	map00140	Steroid hormone biosynthesis	1.31E-07	15
KEGG	map00040	Pentose and glucuronate interconversions	4.02E-07	16
KEGG	map04217	Necroptosis	1.44E-06	11
KEGG	map00053	Ascorbate and aldarate metabolism	3.97E-06	13
KEGG	map00983	Drug metabolism - other enzymes	6.62E-06	16
KEGG	map00860	Porphyrin and chlorophyll metabolism	7.53E-06	13
KEGG	map00830	Retinol metabolism	9.60E-06	13
KEGG	map00760	Nicotinate and nicotinamide metabolism	5.73E-05	6
KEGG	map00790	Folate biosynthesis	6.33E-05	7
KEGG	map00601	Glycosphingolipid biosynthesis - lacto and neolacto series	2.09E-04	4
KEGG	map00310	Lysine degradation	5.96E-04	7
KEGG	map00510	N-Glycan biosynthesis	6.31E-04	6
KEGG	map00590	Arachidonic acid metabolism	1.02E-03	6
KEGG	map00603	Glycosphingolipid biosynthesis - globo and isoglobo series	1.19E-03	4
KEGG	map04146	Peroxisome	2.78E-03	10
KEGG	map01110	Biosynthesis of secondary metabolites	3.63E-03	23
KEGG	map00280	Valine, leucine and isoleucine degradation	1.31E-02	5
BP	GO:0015074	DNA integration	7.46E-30	45
BP	GO:0006310	DNA recombination	1.28E-27	45
BP	GO:0006260	DNA replication	1.64E-06	25
BP	GO:0006334	nucleosome assembly	1.93E-06	9
BP	GO:0006352	DNA-templated transcription, initiation	1.07E-05	9
BP	GO:0050482	arachidonic acid secretion	1.69E-03	5
BP	GO:0006644	phospholipid metabolic process	2.56E-03	5
BP	GO:0006139	nucleobase-containing compound metabolic process	3.75E-03	7
BP	GO:0007608	sensory perception of smell	1.89E-02	6
CC	GO:0000786	nucleosome	9.50E-16	26
MF	GO:0016614	oxidoreductase activity, acting on CH-OH group of donors	3.24E-27	38
MF	GO:0050660	flavin adenine dinucleotide binding	6.21E-26	49

MF	GO:0046982	protein heterodimerization activity	1.29E-12	26
MF	GO:0020037	heme binding	4.31E-10	50
MF	GO:0016705	oxidoreductase activity, acting on paired donors, with incorporation or reduction of molecular oxygen	1.00E-09	45
MF	GO:0004185	serine-type carboxypeptidase activity	1.47E-09	13
MF	GO:0003887	DNA-directed DNA polymerase activity	1.79E-09	26
MF	GO:0005506	iron ion binding	9.79E-09	45
MF	GO:0000166	nucleotide binding	4.25E-05	26
MF	GO:0004499	N,N-dimethylaniline monooxygenase activity	1.68E-04	6
MF	GO:0004497	monooxygenase activity	2.39E-04	11
MF	GO:0003951	NAD+ kinase activity	2.91E-04	6
MF	GO:0008146	sulfotransferase activity	5.69E-04	9
MF	GO:0032440	2-alkenal reductase [NAD(P)] activity	6.17E-04	5
MF	GO:0047522	15-oxoprostaglandin 13-oxidase activity	6.17E-04	5
MF	GO:0004623	phospholipase A2 activity	1.69E-03	5
MF	GO:0050661	NADP binding	6.72E-03	6
MF	GO:0004970	ionotropic glutamate receptor activity	9.89E-03	8
MF	GO:0016627	oxidoreductase activity, acting on the CH-CH group of donors	1.05E-02	7
MF	GO:0004984	olfactory receptor activity	1.89E-02	6
MF	GO:0016758	transferase activity, transferring hexosyl groups	2.05E-02	13

Table S13. GO and KEGG enrichment of the gene family contraction orthologous genes in *P. pectoralis* Hi-C genome Ppec-2.0

Class	ID	Description	p value	Count
KEGG	map02010	ABC transporters	1.83508E-52	37
KEGG	map04976	Bile secretion	9.75962E-40	32
KEGG	map04024	cAMP signaling pathway	5.93217E-26	32
KEGG	map00061	Fatty acid biosynthesis	8.05848E-15	12
KEGG	map04974	Protein digestion and absorption	7.37942E-10	13
KEGG	map04080	Neuroactive ligand-receptor interaction	2.8807E-09	13
KEGG	map04972	Pancreatic secretion	1.81702E-08	13
KEGG	map04910	Insulin signaling pathway	1.29096E-07	12
KEGG	map01212	Fatty acid metabolism	1.96607E-07	12
KEGG	map04152	AMPK signaling pathway	3.34993E-07	12
KEGG	map04540	Gap junction	0.001621345	5
KEGG	map04145	Phagosome	0.002651855	6
KEGG	map04210	Apoptosis	0.009248321	5
KEGG	map04614	Renin-angiotensin system	0.010960343	3
KEGG	map04530	Tight junction	0.019820412	5
BP	GO:0055085	transmembrane transport	3.03462E-15	35
BP	GO:0006508	proteolysis	5.44062E-13	30
BP	GO:0007017	microtubule-based process	1.22017E-05	5
BP	GO:0009058	biosynthetic process	0.000205997	5
BP	GO:0006744	ubiquinone biosynthetic process	0.212113616	1
BP	GO:0015991	ATP hydrolysis coupled proton transport	0.212113616	1
BP	GO:0005975	carbohydrate metabolic process	0.581658551	2
CC	GO:0005874	microtubule	6.67218E-06	5
MF	GO:0042626	ATPase activity, coupled to transmembrane movement of substances	7.16304E-50	34
MF	GO:0016887	ATPase activity	6.50366E-46	39
MF	GO:0008237	metallopeptidase activity	8.83042E-13	17
MF	GO:0004222	metalloendopeptidase activity	8.13463E-12	14
MF	GO:0016740	transferase activity	1.21564E-11	9
MF	GO:0004252	serine-type endopeptidase activity	5.76746E-08	16
MF	GO:0005549	odorant binding	2.19457E-06	12
MF	GO:0005200	structural constituent of cytoskeleton	3.32724E-06	5
MF	GO:0016491	oxidoreductase activity	6.16375E-05	14
MF	GO:0016788	hydrolase activity, acting on ester bonds	6.27702E-05	5
MF	GO:0003824	catalytic activity	0.006921458	14
MF	GO:0005507	copper ion binding	0.025695831	2

MF	GO:0003924	GTPase activity	0.046084832	5
Class	ID	Description	pvalue	Count
KEGG	map02010	ABC transporters	1.83508E-52	37
KEGG	map04976	Bile secretion	9.75962E-40	32
KEGG	map04024	cAMP signaling pathway	5.93217E-26	32
KEGG	map00061	Fatty acid biosynthesis	8.05848E-15	12
KEGG	map04974	Protein digestion and absorption	7.37942E-10	13
KEGG	map04080	Neuroactive ligand-receptor interaction	2.8807E-09	13
KEGG	map04972	Pancreatic secretion	1.81702E-08	13
KEGG	map04910	Insulin signaling pathway	1.29096E-07	12
KEGG	map01212	Fatty acid metabolism	1.96607E-07	12
KEGG	map04152	AMPK signaling pathway	3.34993E-07	12
KEGG	map04540	Gap junction	0.001621345	5
KEGG	map04145	Phagosome	0.002651855	6
KEGG	map04210	Apoptosis	0.009248321	5
KEGG	map04614	Renin-angiotensin system	0.010960343	3

Table S14. GO and KEGG enrichment of the species-specific orthologous genes in *P. pectoralis* Hi-C genome Ppec-2.0

Class	ID	Description	p value	Count
KEGG	map00982	Drug metabolism - cytochrome P450	9.74E-10	24
KEGG	map00980	Metabolism of xenobiotics by cytochrome P450	4.63E-09	24
KEGG	map00983	Drug metabolism - other enzymes	3.02E-07	22
KEGG	map00900	Terpenoid backbone biosynthesis	4.82E-07	12
KEGG	map00140	Steroid hormone biosynthesis	7.48E-07	17
KEGG	map00981	Insect hormone biosynthesis	1.29E-06	13
KEGG	map00830	Retinol metabolism	7.88E-06	16
KEGG	map01110	Biosynthesis of secondary metabolites	1.10E-05	38
KEGG	map00053	Ascorbate and aldarate metabolism	1.31E-05	15
KEGG	map00040	Pentose and glucuronate interconversions	1.55E-05	17
KEGG	map00860	Porphyrin and chlorophyll metabolism	2.63E-05	15
KEGG	map04080	Neuroactive ligand-receptor interaction	1.22E-04	12
KEGG	map04974	Protein digestion and absorption	2.02E-04	11
KEGG	map00480	Glutathione metabolism	7.42E-04	11
KEGG	map04972	Pancreatic secretion	1.77E-03	11
BP	GO:0006508	proteolysis	5.87E-12	59
BP	GO:0008299	isoprenoid biosynthetic process	2.47E-05	8
BP	GO:0006334	nucleosome assembly	3.24E-04	6
BP	GO:0015074	DNA integration	2.02E-03	12
BP	GO:0006310	DNA recombination	4.25E-03	12
CC	GO:0000786	nucleosome	3.70E-11	19
MF	GO:0004252	serine-type endopeptidase activity	7.36E-12	38
MF	GO:0046982	protein heterodimerization activity	1.07E-04	13
MF	GO:0016705	oxidoreductase activity, acting on paired donors, with incorporation or reduction of molecular oxygen	1.53E-04	27
MF	GO:0005549	odorant binding	3.60E-04	19
MF	GO:0005506	iron ion binding	4.73E-04	27
MF	GO:0020037	heme binding	1.44E-03	27
MF	GO:0008425	2-polyprenyl-6-methoxy-1,4-benzoquinone methyltransferase activity	2.04E-03	4
MF	GO:0032440	2-alkenal reductase [NAD(P)] activity	2.04E-03	4
MF	GO:0047522	15-oxoprostaglandin 13-oxidase activity	2.04E-03	4
MF	GO:0004222	metalloendopeptidase activity	3.22E-03	11
MF	GO:0016758	transferase activity, transferring hexosyl groups	5.15E-03	12
